# Metal-Polymer Nanoconjugates Application in Cancer Imaging and Therapy

**DOI:** 10.3390/nano12183166

**Published:** 2022-09-13

**Authors:** André Q. Figueiredo, Carolina F. Rodrigues, Natanael Fernandes, Duarte de Melo-Diogo, Ilídio J. Correia, André F. Moreira

**Affiliations:** 1 CICS-UBI—Health Sciences Research Centre, Universidade da Beira Interior, Av. Infante D. Henrique, 6200-506 Covilhã, Portugal; 2 CPIRN-UDI/IPG—Centro de Potencial e Inovação em Recursos Naturais, Unidade de Investigação para o Desenvolvimento do Interior do Instituto Politécnico da Guarda, Avenida Dr. Francisco de Sá Carneiro, No. 50, 6300-559 Guarda, Portugal

**Keywords:** metallic nanoparticles, metal-polymer nanoconjugates, cancer, photothermal effect

## Abstract

Metallic-based nanoparticles present a unique set of physicochemical properties that support their application in different fields, such as electronics, medical diagnostics, and therapeutics. Particularly, in cancer therapy, the plasmonic resonance, magnetic behavior, X-ray attenuation, and radical oxygen species generation capacity displayed by metallic nanoparticles make them highly promising theragnostic solutions. Nevertheless, metallic-based nanoparticles are often associated with some toxicological issues, lack of colloidal stability, and establishment of off-target interactions. Therefore, researchers have been exploiting the combination of metallic nanoparticles with other materials, inorganic (e.g., silica) and/or organic (e.g., polymers). In terms of biological performance, metal-polymer conjugation can be advantageous for improving biocompatibility, colloidal stability, and tumor specificity. In this review, the application of metallic-polymer nanoconjugates/nanohybrids as a multifunctional all-in-one solution for cancer therapy will be summarized, focusing on the physicochemical properties that make metallic nanomaterials capable of acting as imaging and/or therapeutic agents. Then, an overview of the main advantages of metal-polymer conjugation as well as the most common structural arrangements will be provided. Moreover, the application of metallic-polymer nanoconjugates/nanohybrids made of gold, iron, copper, and other metals in cancer therapy will be discussed, in addition to an outlook of the current solution in clinical trials.

## 1. Introduction

The manipulation of matter at the nanoscale led to the development of various nanomaterials, based on polymers, lipids, ceramics, or metals, that exhibit exciting properties for improving the performance of electronics, medical diagnostics, and therapeutics [1,2]. Particularly, the application of nanoparticles for cancer therapy led to the development of new alternatives from therapeutics (i.e., drug delivery, photothermal and photodynamic effects) to diagnostic and imaging, leveraging the innate ability of these materials to accumulate in the tumor tissue [3,4,5].

In this field, the application of metallic-based nanomaterials (e.g., gold, iron, silver, and copper) has been capturing more attention in recent years due to their characteristic physical (e.g., magnetic behavior, plasmonic resonance, and imaging capacity) and chemical (e.g., radical oxygen species (ROS) generation, and catalytic activity enhancement) properties, which render them as platforms suitable for creating multifunctional cancer therapeutics [2,6,7,8,9]. Moreover, there are several methodologies described for the synthesis of metallic nanoparticles, such as physical or chemical vapor deposition, sol-gel methods, chemical reduction, hydrothermal methods, solvothermal method, laser ablation, and green synthesis processes, allowing the selection of the process most compatible with the available laboratory/industrial conditions and desired physicochemical properties [10,11,12,13]. Additionally, metallic nanomaterials surface functionalization, a strategy often used to refine the therapeutics pharmacokinetics, can be performed by well-known and defined methodologies [14]. Thus, the metallic nanomaterials’ theranostic potential provides an all-in-one solution for cancer diagnosis, therapy, and real-time monitoring, which ultimately can improve the therapeutic outcome of anticancer therapy [15,16]. This is the rationale behind various metallic-based nanomaterials under clinical trials, such as Aurolase^®^, Nanotherm^®^, and Magnablate^®^.

Nevertheless, despite the wide number of publications showing the appealing features of metallic-based nanomaterials, their translation into the clinic is still very limited [17]. Such is widely associated with some toxicological issues, lack of colloidal stability, and the establishment of off-target interactions [18]. Additionally, when subjected to high-energy radiations, the metallic nanoparticles often undergo reshaping processes or are even degraded leading to the loss of their therapeutic potential [19]. Therefore, researchers have been exploring the combination of metallic nanoparticles with other materials, inorganic (e.g., silica) and/or organic (e.g., polymers). Particularly, the combination of the metallic nanoparticles’ physicochemical properties with the superior biological performance of synthetic or natural polymers emerges as a valuable and straightforward approach to develop more effective anti-cancer therapeutics [16,20]. In this review, the application of metallic-polymer nanoconjugates/nanohybrids as a multifunctional all-in-one solution for cancer therapy will be summarized. Initially, the physicochemical properties rendering the metallic nanomaterials’ potential to act as imaging and/or therapeutic agents will be summarized. Then, an overview of the main advantages of metal-polymer conjugation as well as the most common structural arrangements will be provided. Moreover, the application of metallic-polymer nanoconjugates/nanohybrids made of gold, iron, copper, and others in cancer therapy will be discussed, in addition to an outlook of the current solution in clinical trials.

## 2. Metallic Nanoparticles Applications and Therapies

Nanosized metals present optical and electrical properties that differentiate them from other nanomaterials, supporting their application in biomedicine, such as the development of biosensors (e.g., diagnosis of viruses using colloidal gold nanoparticles), bioimaging agents (e.g., iron oxide-based contrast agents), catalysts, mechanical reinforcement, and drug delivery system or other therapeutics. Moreover, these unique characteristics can be explored to create more effective antitumoral nanomedicines. The high density and X-ray attenuation capacity of metallic nanoparticles allow them the intrinsic capacity to be applied as contrast agents for bioimaging applications [21]. Up to now, several studies available in the literature have already shown that metallic nanoparticles provide a higher contrast enhancement in X-ray computed tomography (CT) imaging than the iodine-based contrast agents conventionally used in the clinic [22,23,24,25,26]. On the other hand, nanosized metals, such as gold, silver, and copper, show a pronounced plasmonic resonance phenomenon, i.e., the collective oscillation of the conduction band electrons in metal-based nanomaterials in response to the incident photons [27]. This interaction can lead to light absorption or scattering and is dependent on the size, morphology, distance, and dielectric constant of the metallic nanoparticles and surrounding medium [28,29,30]. In turn, the excited surface electrons can decay to the ground state via different processes (e.g., electron-to-photon, electron-to-electron, and electron-to-phonon energy conversion), the two most common events being the release of the absorbed energy in the form of light or heat [31]. The former is often explored to enhance the quantum efficiency and photostability of fluorophores, allowing the detection of lower quantities of biomarkers used in biosensing or bioimaging [32]. The latter is the foundation stone for the application of metallic nanoparticles in cancer hyperthermia/photothermal therapy [33]. However, it is essential to tailor the nanomaterials to interact specifically with near-infrared (NIR) radiation, a region of the spectra where the major biological components (e.g., collagen, hemoglobin, and water) have the lowest or insignificant absorption [34]. This will reduce the off-target interactions and guarantee a site-specific activation of the metallic nanomaterials. Then, the heat generated by the light-nanoparticles interaction can mediate the destruction of the cancer cells [35,36]. The elevation of the tumor temperature to values superior to 45 °C provokes irreversible damage to cancer cells (e.g., DNA degradation, cell membrane disruption, protein denaturation), leading to cell death (i.e., tumor ablation). Otherwise, if mild temperature increases are achieved (i.e., between 40 and 45 °C), the cell damage is less pronounced and often reversed by the cell repair mechanisms [5,37,38]. Nevertheless, this creates a time window during which the cancer cells are more sensitive to the action of other therapeutic modalities such as chemotherapy [39]. Furthermore, metallic nanomaterials, such as those composed of iron, nickel, and cobalt, can also present magnetic properties, also allowing their application as contrast agents (magnetic resonance imaging (MRI)) and in tumor magnetic hyperthermia [40]. This capacity to be magnetically manipulated by external magnetic fields is also explored to guide these metallic nanomaterials in the human body and promote a tumor-specific accumulation [41,42]. At the tumor site, the utilization of alternating magnetic fields will promote the nanoparticles’ vibration and consequently a localized temperature increase will be obtained [43].

Metallic nanomaterials have also shown the capacity to mediate the formation of ROS [18]. This oxidative stress can influence several cellular processes/structures, e.g., intracellular calcium concentrations, activate transcription factors, induce DNA damage, and lipid peroxidation (cell membrane disruption), and increased amounts of ROS are highly cytotoxic [18]. The mechanism of ROS generation by metallic nanomaterials is influenced by their physicochemical properties (e.g., size, chemical structure, surface area, and charge) [44]. Generally, metallic nanomaterials act as the reactant or catalyst for the reduction of molecular oxygen to water, which yields the production of ROS, such as superoxide radicals and hydroxyl radicals [45]. The ROS generation of metallic nanomaterials can be further boosted by light absorption [46]. During this process, the electrons transit to higher energy bands, facilitating the reaction with water or molecular oxygen and consequently the ROS generation, a process denominated by photodynamic effect [47,48].

Despite the imaging and therapeutic potential of metallic nanoparticles, the in vivo application and translation to the clinic are severely hindered by their low colloidal stability, high reactivity, the formation of the protein corona, and high cytotoxicity [49,50,51]. Therefore, to overcome these limitations, researchers have been combining the superior physicochemical features of metallic nanoparticles with the increased biological properties (e.g., biocompatibility, enhanced blood circulation time, targeting capacity) of synthetic and natural polymers, often referred to as metal-polymer nanocomplexes or nanohybrids.

## 3. Metal-Polymer Based Nanomaterials

**Figure 1:**
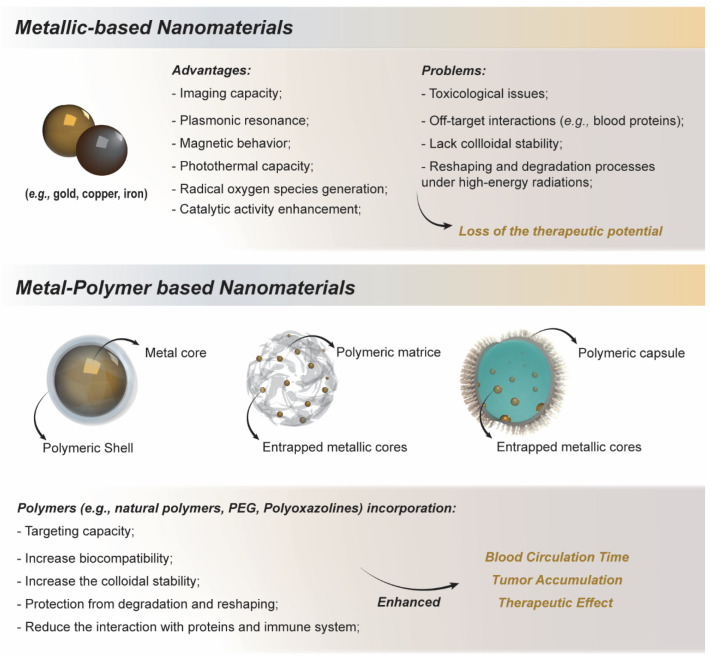
Overview of the properties of metallic nanoparticles, advantages of the polymers’ inclusion, and representation of the most common structural organizations of the metal-polymer nanohybrids/complexes.

The metal-polymer nanocomplexes or nanohybrids are a class of nanomaterials that aim to address the biological bottlenecks of the metallic nanoparticles’ administration in humans. For that purpose, several strategies have been explored to create these new nanomaterials (Figure 1), namely the (i) surface coating of metallic cores (e.g., physical linkage and layer-by-layer), (ii) entrapment of metallic cores within polymeric matrices (e.g., nanoparticle in situ growth or post-synthesis entrapment), and (iii) the utilization of polymeric capsules [52,53,54]. One of the main rationales behind the introduction of polymers is to increase the colloidal stability of metallic nanoparticles and avoid protein adsorption during the nanoparticles’ circulation in the blood [55,56].

Highly hydrophilic polymers are often associated with improvements in the nanoparticles’ blood circulation time, namely by minimizing the nanoparticle-protein interaction, avoiding nanoparticles’ aggregation as well as the size and charge changes, and reducing the recognition by the immune cells [57,58]. For this purpose, polymers, such as poly(ethylene glycol) (PEG), poly(oxazolines) (POX), and poly(zwitterion)s, have been excelling in enhancing the nanoparticles’ blood circulation [59]. The PEG and POX anti-fouling properties, which are attributed to two main events, (i) the steric repulsion and (ii) the water barrier, impede the nanoparticles–proteins interaction, avoiding the formation of a protein corona that negatively impacts the nanomaterials’ biological performance [60,61]. In turn, the zwitterionic polymers present overall electrostatic neutrality and high chain hydration that confer to them a stealthing capacity [62,63]. The higher blood circulation times associated with the utilization of these polymers will increase the nanomaterials’ probability to accumulate and interact with the tumor cells, which can be essential for achieving superior antitumoral effects [64]. On the other hand, the natural and synthetic polymers can also imprint a stimuli-responsive (e.g., pH, temperature, and enzymatic) behaviour on the metallic-based nanomaterials, which can be particularly advantageous for controlling drug delivery [33,65,66,67,68]. In this regard, the heat generated by the nanomaterials (e.g., PTT and magnetic hyperthermia) can be also explored to induce phase changes in the polymers (e.g., Poly(N-isopropylacrylamide) (PNIPAM)) or increase their solubility, which will trigger the drug release [68,69]. Additionally, at the tumor site, the metal-polymer nanocomplexes/nanohybrids will also have an impact on the physiological conditions that can be explored to trigger the drug release, such as an acidic pH, overexpression of certain enzymes (e.g., matrix metalloproteinases), and an increased RedOx environment [65,67,70]. Apart from the passive accumulation of the nanomaterials at the tumor site, usually dependent on the enhanced permeability and retention (EPR) effect, the polymers or other targeting moieties can confer a specific recognition of molecules overexpressed in the tumor tissue [5,71]. This higher specificity towards cancer cells will favor the accumulation of nanomaterials in these areas as well as the nanomaterials–cancer cell interaction [72]. Therefore, the metal–polymer nanocomplexes or nanohybrids show the potential to create novel and more effective anticancer therapeutics. In the following section, the application of metallic–polymer nanoconjugates/nanohybrids made of gold, iron, copper, and others in cancer therapy will be overviewed, showing the therapeutic modalities that each metal nanoparticle allows to explore.

### 3.1. Gold-Polymer Conjugates

Gold nanoparticles are one of the most explored metallic nanomaterials for biomedical applications, from bioimaging to drug delivery [73,74,75]. The applicability of gold nanomaterials in bioimaging is demonstrated in different works in the literature, principally as a contrast agent for CT imaging [76]. For example, Xi and co-workers observed that, when an X-ray beam of 100 KeV is used, gold nanoparticles present an absorption coefficient two-times superior to that obtained with iodine (a conventional contrast agent used in the clinic) [77]. In turn, the pronounced surface plasmon resonance of gold nanoparticles has been supporting the development of novel photothermal therapies for cancer therapy (Table 1). This phenomenon can be fine-tuned to enhance the gold nanoparticles’ absorption in the NIR region (700–1100 nm) by optimizing the nanoparticles’ size and/or shape (e.g., spheres, cages, stars, and rods) [37,78]. Per se, the application of gold nanospheres in cancer photothermal therapy (PTT) is limited since its absorption peak is in the 500–550 nm region [79]. However, the organization of gold nanospheres in nanoclusters or shells renders a shift in the absorption spectra to the NIR region [2,5]. This change in the gold nanoparticles’ optical properties is attributed to the coupling of the plasmon resonance of adjacent gold nanospheres [80]. Therefore, the fine-tuning of the gold nanoclusters or shells’ absorption spectra can be achieved by optimizing the nanospheres’ size and interparticle distance [2]. In turn, the surface plasmon resonance in non-spherical gold nanoparticles varies with the nanoparticle surface [81,82]. For example, the two different surfaces in gold nanorods, longitudinal and transversal surfaces, originate two absorption bands. The transversal surface leads to an absorption band in the 500–550 nm region of the spectra, whereas the longitudinal surface originates an absorption peak that can be fine-tuned from the visible to the NIR region of the spectra [83,84]. This peak generated by the longitudinal surface resonance is determined by the aspect ratio of gold nanorods (i.e., quotient between length and width) [85]. Otherwise, the surface plasmon resonance of gold nanostars is defined by the particle’s core size, the number of tips, as well as the tips’ length and width [2]. Therefore, the gold nanoparticles’ plasticity and fine-tunning ability to present a high absorption efficiency in the NIR region of the spectra propelled their application in the cancer PTT. Nevertheless, despite the theragnostic potential of gold nanoparticles, their direct application in the human body is hindered by their high affinity to establish interactions with thiol groups, which favors the interaction with different biomolecules and lead to the nanoparticles’ aggregation [80]. Moreover, the gold nanoparticles can also be degraded when exposed to high-energy radiations, such as those used in CT imaging and PTT, causing the loss of their bioactivity [86]. Furthermore, several reports in the literature also describe that gold nanoparticles are strongly accumulated in the kidneys, causing nephrotoxicity, and may also trigger the lysis of red blood cells [78]. To address these issues, researchers have been exploring the development of gold-polymer nanocomplexes or nanohybrids to increase colloidal stability, biocompatibility, and even tumor specificity.

**Figure 2:**
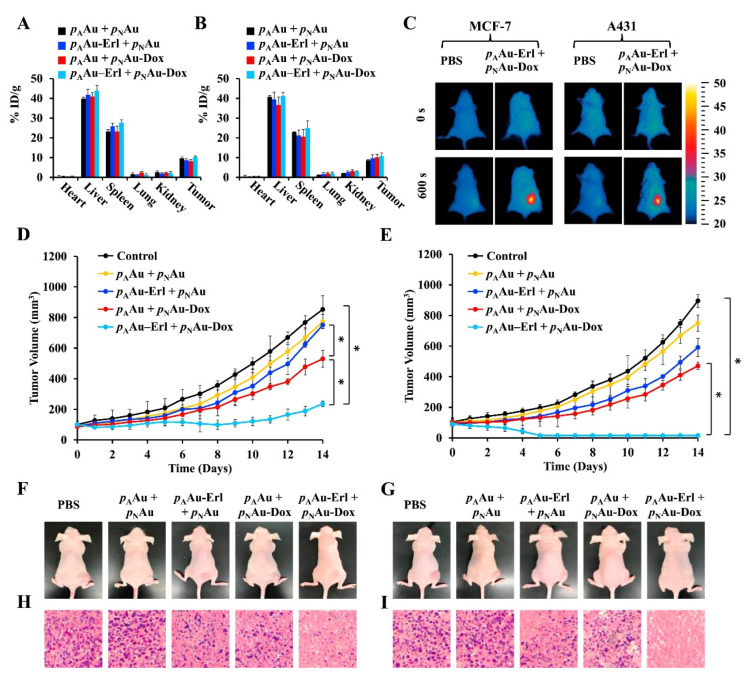
In vivo evaluation of the p_A_Au-Erl plus p_N_Au-Dox antitumoral efficacy. Biodistribution analysis of p_A_Au + p_N_Au, p_A_Au-Erl + p_N_Au, p_A_Au + p_N_Au-Dox, and p_A_Au–Erl + p_N_Au-Dox (12.4 Au mg kg^−1^ of mouse, Erl:Dox = 1:1) in MCF-7 (**A**) and A431 (**B**) tumor-bearing mice. Infrared thermal images of MCF-7 and A431 tumor-bearing mice before and after NIR laser irradiation (808 nm, 0.5 W cm^−2^, for 10 min) (**C**). Analysis of the tumor growth curve in MCF-7 (**D**) and A431 (**E**) tumor-bearing mice. * *p* < 0.05 analysed by ANOVA. Photos and histological analysis of MCF-7 (**F**,**H**) and A431 (**G**,**I**) tumor-bearing mice. Reprinted with permission from [68]. Copyright (2019) Elsevier.

Peng and colleagues demonstrated the applicability of gold-based nanomaterials in bioimaging by following the biodistribution and tumor accumulation of PEGylated dendrimer-entrapped gold nanospheres [91]. In fact, the authors reported that these nanomaterials have an attenuation intensity higher than Omnipaque (an iodine-based contrast agent), which allowed them to follow the PEGylated dendrimer-entrapped gold nanoparticles in the blood circulation, after intravenous injection, as well as to perform the CT imaging of SPC-A1 xenograft tumors. Moreover, the authors reported that the PEGylated dendrimer-entrapped gold nanoparticles had a half-decay time in the blood circulation of 31.76 h, which was 2.5 times higher than that of bare gold nanorods previously reported. Furthermore, Gu et al. produced RGD-modified mPEG-PLGA nanocapsules containing gold nanoclusters and indocyanine green for the imaging and PTT of breast cancer [92]. The loaded mPEG-PLGA nanocapsules were formed via a water-in-oil-in-water emulsion using sonication, where (i) the first emulsion consisted of the water phase with gold nanoclusters and the oil phase with indocyanine green and the mPEG-PLGA. In the second emulsion, the mPEG-PLGA nanocapsules were further modified with poly(vinyl alcohol) and poly(acrylic acid), allowing the subsequent functionalization with RGD peptide via carbodiimide chemistry. The authors demonstrated that both one-photon and two-photon imaging techniques could be used to follow the nanocapsules in 4T1 tumor-bearing BALB/c mice. Moreover, the RGD-modified nanocapsules showed a preferential accumulation in U87-MG cancer cells (overexpressing ανβ_3_ integrins), when compared to MCF-7 cancer cells (low expression of ανβ_3_ integrins), leading to the almost complete ablation of the cancer cells after irradiation with a NIR laser (808 nm, 2 W cm^−2^, for 5 min). Feng and co-workers developed two different tumor-targeted gold nanocages for the combinatorial chemo-PTT of breast cancer [68]. For that purpose, pH-responsive gold nanocages were formulated via electrostatic interaction between the poly(acrylic acid) and the surface of the particles, entrapping the gold particles in the polymeric chains (p_A_Au nanoparticles) and loaded with Erlotinib (Erl), an epidermal growth factor receptor (EGFR) inhibitor. In turn, temperature-responsive gold nanocages were produced by reacting them with the thiol-terminated N-isopropylacrylamide (NIPAM) and acrylamide (AM) (p(NIPAM-co-AM) co-polymer (p_N_Au nanoparticles) and subsequently loaded with doxorubicin (Dox). The p_A_Au nanoparticles showed a pH-triggered Erl release due to the protonation of poly(acrylic acid) in acidic pH (i.e., loosening of the polymeric barrier), 4.5%, 24.8%, 44.1%, or 66.3% Erl released after 6 h at pH 7.4, 6.5, 6, or 5. Otherwise, the Dox-loaded p_N_Au nanoparticles were responsive to the irradiation with a NIR laser (808 nm, 0.5 W cm^−2^, for 10 min) and consequent increase in temperature (i.e., superior to 45 °C, a value higher than the lower critical solution temperature). The authors reported 46.2% of Dox released in 10 h, after a 10 min NIR laser irradiation, contrasting with the 5% detected in the absence of NIR irradiation. The in vivo studies performed in MCF-7 and A431 tumor-bearing mice demonstrated a passive and preferential accumulation in the tumor tissue (Figure 2). Moreover, the combinatorial therapy led to the reduction of A431 tumors’ size by 98%, after 14 days, whereas in MCF-7 tumors (low expression of EGFR), these nanomaterials only slowed the tumor growth.

### 3.2. Iron-Polymer Conjugates

**Figure 3:**
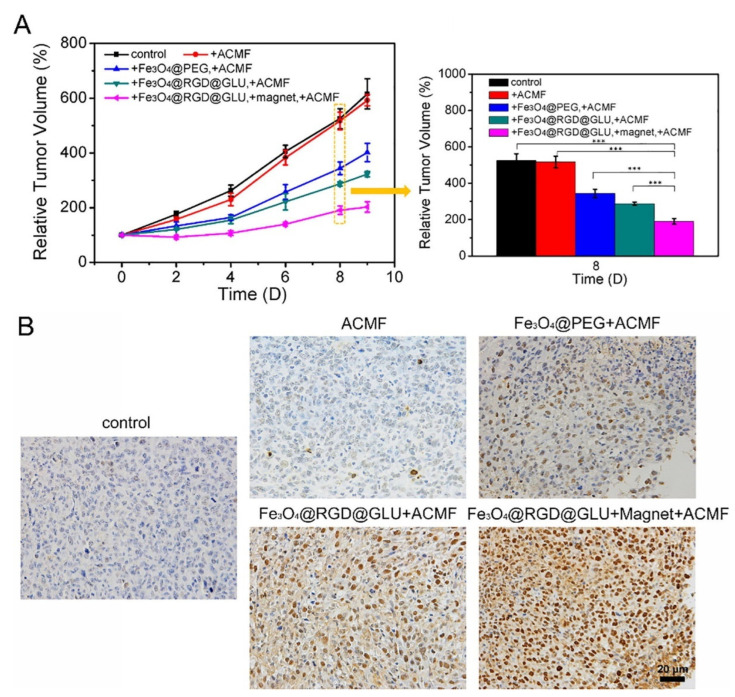
Analysis of the tumor growth curves for 10 days after treatment with iron oxide nanomaterials, mean ± SE and *n* = 5 (**A**). TUNEL histological analysis of mice tumors at day 9. Statistical significance is represented by the asterisk (*** *p* < 0.001). TUNEL assay for mice tumors on day 9 following various treatments (**B**). Reprinted with permission from [105]. Copyright (2019) Elsevier.

The utilization of iron oxide nanoparticles can be explored in different biomedical applications, such as drug delivery, hyperthermia, and magnetic resonance imaging. Similar to gold nanoparticles, iron oxide can be produced in different shapes, such as spherical, rod-like, and cubical [93,94]. These nanoparticles (Fe_2_O_3_ and Fe_3_O_4_), when their size is inferior to 20 nm, present a superparamagnetic behavior at room temperature, i.e., the magnetization of the nanoparticles is close to 0 in the absence of an external magnetic field [95,96]. The iron oxide nanoparticles magnetism allows for widespread application in cancer therapy (Table 2), namely as contrast agents for magnetic resonance imaging: Feridex^®^, Resovist^®^, and Endorem^®^. The data available in the literature indicate that iron oxide nanoparticles are less toxic than the conventionally used gadolinium-based contrast agents, without presenting significant losses in imaging capacity [95]. Moreover, the magnetic properties of iron oxide nanoparticles have also been explored to direct the accumulation of the nanoparticles, specifically towards the tumor tissue, and in certain cases mediate a hyperthermic effect [97,98]. The former explores the application of an external magnetic field to guide the nanoparticles and promote their accumulation in the target tissue [99]. The latter employs an alternating magnetic field to induce the oscillation of iron oxide nanoparticles, which in turn generate heat [97]. This hyperthermic effect is dependent on the magnetic properties of the nanoparticles as well as on the frequency and intensity of the alternating magnetic field [100,101]. However, the biological application of iron oxide nanoparticles is hindered by their limited colloidal stability, tending to agglomerate when in contact with biological fluids [102]. With this in mind, Xu et al. prepared GSH-responsive hyaluronic acid-coated small iron oxide nanoparticles (HIONPs) for the diagnosis of liver metastases [103]. The nanoparticles were formed via a one-pot method, promoting the oxidation of ferrous ions to create iron oxide nanoparticles that were coated with hyaluronic acid modified with dopamine through the establishment phenol−metal coordination interactions. The in vitro measurements in a 0.52 T NMR instrument showed that the HIONPs have a longitudinal proton relaxivity (*r_1_*) of 41.3 Fe mM^−1^ s^−1^, indicating the applicability of HIONPs as *T_1_* MRI contrast agents. This was confirmed using a 3 T MR scanner where the HIONPs led to higher *T_1_*-weighted magnetic resonance signals and lower *T_2_*-weighted magnetic resonance signals when the Fe concentration increased from 0 to 0.2 mM. Moreover, the application of HIONPs (Fe—0.03 mmol kg^−1^) in mice with B16F10, 4T1, and CT26 liver metastases allowed the metastases detection via MRI, showing the highest contrast-to-noise ratio 1 h after injection. This capacity is attributed to the higher GSH concentration in the mice liver tissue (i.e., 11 to 76-times higher) when compared to the tumor/metastases. The higher GSH concentration promotes the removal of the hyaluronic acid coating and consequent nanoparticle aggregation, which led to a significant decrease in the *r_1_* value. In this way, the hepatic tissues became dark whereas the tumor/metastases are bright in the MRI. Moreover, the authors also described that the HIONPs presented a higher imaging capacity than Primovist^®^ (Gd-based imaging agent), where the metastases and surrounding liver tissue presented a similar contrast-to-noise ratio. In another work, Xiao and co-workers developed ultrathin vesicles with multimodal imaging capacity for the combinatorial chemo-PTT of cancer [104]. For that purpose, ultrasmall superparamagnetic iron oxide nanoparticles, cisplatin, and liquid perfluorohexane were encapsulated in PLGA nanovesicles, followed by the formation of an ultrathin silica layer to prevent leakage. Then, the surface of the particles was modified with polyaniline (a photothermal agent) and functionalized with R8-RGD. In these nanoparticles, the iron oxide acted as a *T_2_*-weighted magnetic resonance contrast agent, with a transverse relaxivity (*r*_2_) value of 258.5 Fe mM^−1^ s^−1^, three times superior to the iron oxide nanoparticles alone. Moreover, the in vivo studies in A549 tumor-bearing mice showed that the administration of the PLGA vesicles containing the iron oxide nanoparticles allows the monitoring of the changes in the tumor cellularity via MRI. Additionally, the chemo-photothermal combinatorial treatment led to a significant regression, close to 97%, of the tumor volume in 21 days. Chen and colleagues produced a dual-targeted magnetic iron oxide nanoparticle for the imaging and hyperthermia of breast tumors (Figure 3) [105]. The iron oxide nanoparticles were coated with a DSPE-PEG2000 shell and then modified with RGDyK (neovascular endothelium targeting) and D-glucosamine (glucose transporter affinity) via carbodiimide chemistry. The authors observed that the combination of magnetic and active targeting approaches resulted in the best contrast effect on *T_2_*-weighted MRI from 3 to 48 h, showing the tumor region completely dark due to the accumulation of the nanoparticles. The tumor/normal tissue signal ratio at 48 h for active targeting strategies was 0.6 whereas for the magnetic plus active targeting combination this value was inferior to 0.3, showing a higher difference in the signal obtained in the tumor and normal tissues. Moreover, the utilization of alternating current magnetic fields (1.485 × 10^9^ Am^−1^ s^−1^), focused on the tumor region, induced a temperature increase to 44 °C, which successfully slowed the growth of 4T1 tumors when compared to the control group (i.e., the relative tumor volume of 500% and 200% for control and iron oxide groups, respectively).

### 3.3. Copper-Polymer Conjugates

**Figure 4:**
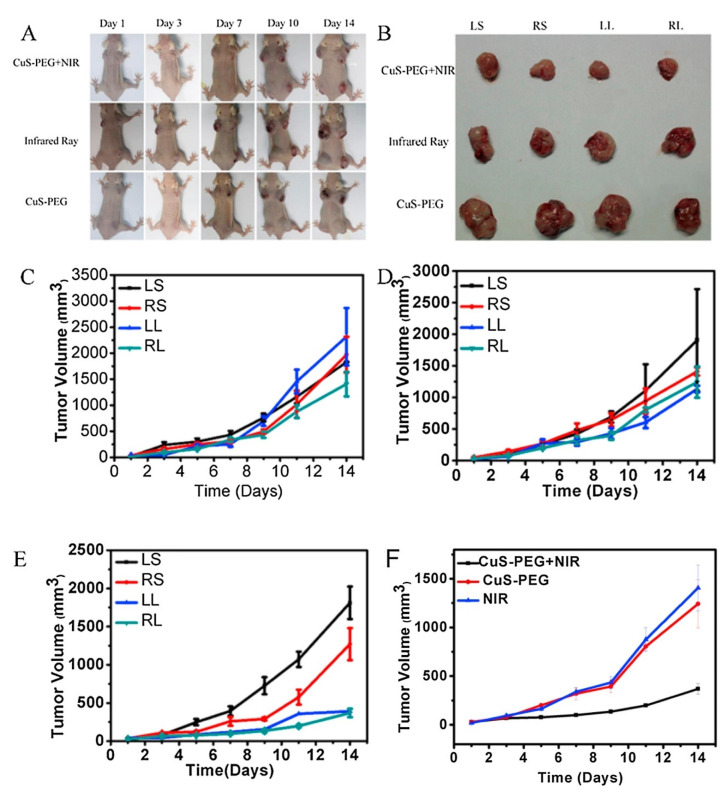
Photos of the tumor-bearing mice at 1, 3, 7, 10 and 14 days (**A**) and SPC-A-1 tumors at day 14 (**B**) after the treatment of the nanomaterials. Tumor growth curves in the control group; 0 µL CuS-PEG in LS, 30 µL CuS-PEG in the RS, 50 µL of CuS-PEG in the LL, and 100 µL CuS-PEG in the RL (**C**). Tumor growth curves in the NIR—irradiated group; 0 µL NS in the LS, 30 μL NS in the RS, 50 μL NS in the LL, and 100 μL NS CuS-PEG in the RL (**D**). Tumor growth curves in the CuS-PEG + NIR group, 100 μL NS in the LS, 30 μL CuS-PEG in the RS, 50 μL CuS-PEG in the LL, and 100 μL CuS-PEG in the RL (**E**). Tumor growth volume curve comparison for 100 μL (30 μg/mL) CuS-PEG +NIR in the RL; 100 μL (30 μg/mL) CuS-PEG in the RL, and 100 μL NS in the RL (**F**). LS—left shoulder administration; RS—right shoulder administration; LL—left leg administration; RL—right leg administration; NS—normal saline solution. Reprinted with permission from [111]. Copyright (2017) Elsevier.

Copper nanomaterials have emerged in recent years as promising inorganic nanoparticles for biomedical applications (Table 3). Copper is a transition metal and can be engineered to form various nanomaterials, such as copper oxides, copper selenides, and copper sulfides [111,112,113]. Among them, copper sulfides have been the most explored due to the simple synthesis process and high NIR absorbing capacity, allowing their application in cancer PTT [37]. On the other hand, the copper nanomaterials can also be used as Fenton-like reagents mediating the formation of ROS (chemodynamic therapy) [114]. Moreover, the generation of ROS can also be stimulated under light irradiation (photodynamic therapy (PDT)) [115]. However, copper is often defined as more toxic than iron and gold, which makes the release of copper ions in the human body undesirable [116,117]. With that in mind, Li et al. showed that the surface functionalization with an amphiphilic polymer, poly(isobutylene-alt-maleic anhydride) (PMA), enhances the colloidal stability of copper telluride nanoparticles without visible agglomeration for periods longer than one month [118]. Furthermore, in vitro studies performed in 3T3 fibroblasts showed significant cytotoxicity after irradiation with a NIR laser (830 nm, 0.5 mW cm^−2^, for 2 s). In another work, Li and colleagues developed a PEGylated copper sulfide nanoparticle for the simultaneous PDT and PTT of lung cancer (Figure 4) [119]. For that purpose, thiolated-PEG was reacted with the copper sulfide nanoparticles rendering the PEGylated nanoparticles. The authors observed that the nanoparticles’ irradiation (30 µg mL^−1^) with a NIR laser (808 nm, 1 W cm^−2^, for 10 min) reaches temperatures superior to 42 °C. Moreover, the authors also observed the continuous quenching of the p-nitrosodimethylaniline (RNO) absorption under NIR irradiation, indicating the generation of ROS during this period. Moreover, the in vivo studies in SPC-A-1 tumor-bearing mice showed that the combination of PDT and PTT decreases tumor growth, observing a 5% increase in the tumor volume at day 14 after administering PEGylated copper sulfide nanoparticles (30 µg mL^−1^) plus NIR. Similarly, Shi et al. produced a PEGylated copper sulfide nanoparticle modified with RGD to target metastatic gastric cancer [120]. The nanoparticles were formed by the reaction of thiolated-PEG-COOH and copper sulfide, followed by the RGD modification using carbodiimide chemistry. The obtained nanoparticles showed computed-tomography contrast capacity similar to the Iodixanol (a clinically used contrast agent). Furthermore, the irradiation of the nanoparticles (60 µg mL^−1^) with a NIR laser (808 nm, 1 W cm^−2^, for 5 min) led to a temperature increase to 60 °C. In the in vivo studies, the authors observed that the RGD-modified PEGylated copper sulfide nanoparticles allowed the identification of MKN45 tumors and metastasis in sentinel lymph nodes through *T_2_*-weighted magnetic resonance images. Moreover, the NIR laser irradiation (808 nm, 1 W cm^−2^, for 10 min) promoted the increase in tumor/metastases temperature to 57 °C, leading to the complete ablation of the metastatic MKN45 tumors (sentinel lymph nodes weight similar to the healthy ones, 2.5 mg).

### 3.4. Other Metal-Polymer Nanoconjugates

Apart from the previously presented gold-, iron-, and copper-based nanomaterials, other metals have also been explored to create novel and more effective anticancer therapeutics (Table 4). Zinc-based nanoparticles, particularly zinc oxide (ZnO), are considered relatively biocompatible and generally regarded as safe. These nanoparticles present photoluminescence properties and a band gap that facilitates the interaction with oxygen and hydroxyl ions prompting the generation of superoxide and hydroxyl radicals (Table 4) [130,131]. Moreover, this ROS generation shows a certain selectivity towards cancer cells, decreasing the potential for inducing side effects [132]. Song and co-workers functionalized ZnO nanoparticles with polyvinylpyrrolidone for application in the imaging and therapy of colon cancer [133]. These authors reported that the surface functionalization maintained the stability of nanoparticles for 14 days, whereas non-coated ZnO nanoparticles aggregated after three days, without impacting the ROS generation capacity. Moreover, the authors also observed that the administration of the polyvinylpyrrolidone coated ZnO nanoparticles, at a concentration of 50 µg mL^−1^, reduced the viability of SW480 cancer cells to 54% due to ROS generation. In turn, the ROS generation was boosted by the irradiation with UV light, with cell viability of 15% at a concentration of 50 µg mL^−1^ (IC_50_ of 21.688 µg mL^−1^). This PDT capacity was also observed in the SW480 tumor-bearing mice, where the nanoparticles plus light treatment slowed the tumor growth for 28 days, a tumor inhibition rate of 61.1%.

Platinum (Pt) is a catalytic noble metal that has been explored in cancer therapy, namely in the form of chemotherapeutic drugs containing platinum atoms, such as cisplatin and derivatives [134]. The utilization of platinum nanoparticles is focused on the release of platinum ions that will induce DNA damage and provoke cell death [135]. Additionally, the platinum nanoparticles can also mediate the generation of ROS or act as photothermal agents [136,137]. Chen and colleagues developed PEGylated porous platinum nanoparticles loaded with Dox for application in breast cancer therapy [138]. In the synthesis process, Pluronic F127 was used as a surfactant for the platinum nanoparticles and then reacted with thiolated-mPEG. This surface modification enhanced the solubility and stability of the platinum nanoparticles. Moreover, the authors also reported that the presence of a 10 mHz square wave AC field (10 mA) further enhanced the ROS generation capacity. In turn, the combination of Dox delivery and ROS generation induced the regression of 4T1 tumors, showing a tumor growth inhibition of 95.5% after 14 days. Zhu and co-workers prepared sodium hyaluronate stabilized platinum nanoparticles (HA/Pt) for mediating a photothermal effect [139]. Apart from the nanoparticle stabilization, the HA functionalization increased the nanoparticles’ specificity towards the MDA-MB231 cancer cells (overexpression of CD44), when compared to the uptake by NIH3T3 cells (low expression of CD44). Furthermore, the in vivo studies in MDA-MB231 tumor-bearing mice revealed that upon irradiation with a NIR laser (808 nm, 1 W cm^−1^, for 10 min), the HA/Pt nanoparticles induced an increase in the temperature of the tumor to 44 °C, which translated to a reduction in the tumor growth for 14 days.

Silver (Ag) is a noble metal with vast applications, being widely applied in the biomedical field as an antimicrobial agent [140]. The silver nanoparticles can mediate ROS formation and consequently induce lipid peroxidation, protein oxidation, and DNA damage [141,142]. Park and co-workers developed indocyanine green-loaded silver nanoparticles functionalized with PEG and BSA for application in cancer PTT [143]. The BSA was used to stabilize the produced silver nanoparticles, followed by the reaction with NHS-PEG for obtaining the functionalized silver nanoparticles. The authors reported that the PEG-BSA silver nanoparticles were stable in solution for at least five days after the synthesis. Moreover, the combination of indocyanine green-silver nanoparticles resulted in a more stable photothermal effect, reaching temperatures of ≈45 °C even after three irradiations with a NIR laser (885 nm, 1.3 W, for 10 min). The in vivo studies performed in B16F10 tumor-bearing mice showed a preferential accumulation in the liver, kidney, and tumors and upon irradiation (885 nm, 0.95 W for 20 min), the tumors’ temperature reached 49 °C. This photothermal effect led to the tumors’ ablation after four days.

### 3.5. Clinical Trials

Since the 1990s, more than 50 nanomedicines have been approved by the Food and Drug Administration (FDA) and are currently on the market [151]. However, in the last decade, only a small number of formulations successfully reached the clinic for cancer treatment, a fact that is in part related to the poor pharmacokinetics of the nanomaterials [152]. The latest data indicate that less than 1% of the administered nanoparticles reach the tumor site [153]. Nevertheless, most of the approved nanomedicines are based on liposomes, protein nanoparticles, nano-emulsions, and metal oxide nanoparticles.

Particularly, iron oxide nanoparticles’ clinical utility is demonstrated by the FDA approval for application in cancer diagnosis, hyperthermia, and iron deficiency anemia. Among them, it is worth highlighting the various contrast agents based on iron oxide nanoparticles commercially available for MRI, such as Feridex^®^, Resovist^®^, and Endorem^®^. Moreover, there are more systems under clinical trial, such as Magnablate^®^ and Nanotherm^®^ (Table 5). The former is an iron oxide nanoparticle developed for magneto-hyperthermia applications that underwent a Phase 0 clinical trial (ClinicalTrials.gov Identifier: NCT02033447), with 12 participants, for the thermoablation of prostate cancer (no results are yet available) [154]. In turn, Nanotherm^®^ is also based on an aqueous suspension of iron oxide nanoparticles and was already approved by the European Medicines Agency (EMA) for brain tumor treatment [37]. Moreover, recently, a new clinical trial (ClinicalTrials.gov Identifier: NCT05010759; still recruiting) was announced to study the application of Nanotherm^®^ in the ablation of prostate carcinoma.

Regarding gold-based nanoparticles, to this date, two promising nanomedicines are currently in clinical trials [151]. AuroLase^®^ uses the particles denominated as AuroShell^®^, a PEGylated silica-gold nanoshell with ≈150 nm in size, for the laser-activated thermal ablation of solid tumors, metastatic lung tumors, and cancer prostatic tissue [151,155]. In the clinical trial (ClinicalTrials.gov Identifier: NCT01679470), a single dose of AuroShell^®^ was administered to promote the ablation of primary and/or metastatic lung tumors, still, to this date, no results were posted [156]. There is a second clinical trial (ClinicalTrials.gov Identifier: NCT00848042) involving the utilization of this nanomedicine for the treatment of patients with refractory and/or recurrent head and neck tumors. The patients were subjected to one or more doses of laser irradiation (808 nm) [30,33]. The participants were divided into three groups: (i) five participants, Auroshell^®^ dose 4.5 mL kg^−1^, laser potency 3.5 W; (ii) five participants, Auroshell^®^ dose 7.5 mL kg^−1^, laser potency 4.5 W; (iii) one participant, Auroshell^®^ dose 7.5 mL kg^−1^, laser potency 5 W. However, no data were published to assess the effect on the targeted tumors. In a more recent clinical trial (ClinicalTrials.gov Identifier: NCT02680535), the Auroshell^®^ nanoparticles were tested in combination with the MRI/Ultrasound fusion technology to promote the focal ablation of neoplastic prostate tissue. The extension of this clinical trial is still active (ClinicalTrials.gov Identifier: NCT04240639), however no data have been found describing the evolution of the targeted tumors. The NU-0129^®^ is another gold-based nanomedicine under clinical trial (ClinicalTrials.gov Identifier: NCT03020017). This nanoparticle is formed by a spherical gold nanoparticle conjugated with siRNA oligonucleotides for targeting BCL2L12 oncogene in glioblastoma multiforme or gliosarcoma treatment applications [151,157]. The early results showed that the NU-0129^®^ nanoparticles can cross the blood–brain barrier without unexpected adverse effects, still pending the data regarding antitumoral efficacy [158].

## 4. Conclusions

Metallic-based nanomaterials have been showing promising results in a variety of cancer-related applications, from imaging to the ablation of tumors. Nevertheless, several of these nanomedicines remain in a preclinical stage, an exception being the application of iron oxide-derived nanomaterials in the imaging of tumors.

In this review, the unique set of physicochemical properties that make the metallic nanoparticles highly promising for biomedical applications is described. The high density and X-ray attenuation capacity make these nanomaterials natural contrast agents for conventional imaging techniques such as CT. Moreover, the surface plasmon resonance and/or the magnetism allow the utilization of hyperthermia treatments (e.g., PTT and magneto-hyperthermia). Therefore, the conjugation with natural and/or synthetic polymers can further increase the biological performance of the metallic nanoparticles by enhancing the colloidal stability and blood circulation time as well as conferring additional specificity to the cancer cells. Nevertheless, there remain significant challenges to overcome and effectively translate the metallic-polymer nanoconjugates/nanohybrids to the clinic. Additional studies on the biosafety and long-term fate of these nanomaterials are still missing. Moreover, the optimization and scale-up of the production methods are mandatory to decrease batch-to-batch variability. Furthermore, regulatory agencies should create a comprehensive set of guidelines for the translation of metallic nanoparticles to the clinic.

In summary, the metallic-polymer nanoconjugates/nanohybrids have the potential to support the development of more effective and multifunctional all-in-one nanomedicines, with the capacity to diagnose and treat the tumor as well as monitor in real-time its response to the treatment. Such raw potential of metallic-polymer nanoconjugates/nanohybrids presents an area of opportunity for both researchers and industrial partners (e.g., pharmaceutics) to develop a new generation of nanomedicines for cancer therapy. Moreover, it is worthwhile to notice that the physicochemical properties of metallic-polymer nanoconjugates/nanohybrids can also be explored to create innovative solutions in the biomedical field, such as biosensors, antimicrobial agents, and tissue regeneration.

## Figures and Tables

**Table 1:** Gold-based metallic-polymer nanoconjugates/nanohybrids, their physicochemical properties, and therapeutic applications (N.D.—non disclosed, N.A.—not applicable).

Metal	Morphology	Modification	Size (nm)	Surface Charge (mV)	Loading	In Vitro	In Vivo	Application	Ref.
Gold	Rods	UCST polymer (P(AAm-co-AN)-DDAT), metalloproteinase 2 (MMP-2)-sensitive peptides	Length ≈ 48.04; Width ≈ 12.08	N.D.	Doxorubicin (DOX)	HepG2 cells	HepG2 tumor-bearing mice	PTT (λex = 808 nm) and chemotherapy	[33]
		Mesoporous silica; D-α-Tocopherol polyethylene glycol 1000 succinate (TPGS), and Hyaluronic acid (HA)	Length ≈ 85; Width ≈ 64	−3 ± 5 and −10 ± 4 for TPGS/HA ratios of 1:1 and 4:1, respectively	N.A.	HeLa cells	N.A.	PTT (λex = 780 nm)	[86]
		Mesoporous silica, HA, and polyethyleneimine (PEI)	Length: 88 ± 5; Width: 63 ± 5;	−10 ± 2	Acridine Orange (AO)	HeLa cells	N.A.	PTT (λex = 750 nm) and chemotherapy	[87]
	Spheres	Poly(ethylene glycol) (PEG) and Lactofferin (LF)	5	N.D.	N.A.	Caco-2, U87MG cells	GBM tumor-bearing mice	PTT (λex = 532 nm)	[54]
	Stars	Polydopamine (PDA) and Folic acid (FA)	149 ± 3	−19 ± 2.7	DOX	MCF-7, MCF-7/ADR, NIH/3T3, and HaCaT cells	MCF-7/ADR bearing mice	PTT (λex ≈ 800 nm) and chemotherapy	[56]
		Dendritic polyglicerol (dPG) and HA	68.1	13.9	Retinoic acid (RA)	MDA-MB-231 cells	4T1 tumor-bearing mice	PTT (λex ≈ 800 nm) and chemotherapy	[88]
		PEG and CD133 antibody	≈120	−22.47	IR780/DTX	PC3 cells	PC3 tumor-bearing mice	PTT (λex = 810 nm), PDT, and chemotherapy	[89]
	Cages	Poly (acrylic acid) (p_A_) or Poly(NIPAM-co-AM) (p_N_)	for p_A_(Au) ≈130; N.D. for p_N_(Au)	≈−4 for p_A_(Au) formulation at pH 7.4; N.D. for p_N_ (Au) formulation	p_A_(Au)-loaded with Erl and p_N_(Au) loaded with DOX	A431 or MCF-7 cells	A431 or MCF-7 tumor-bearing mice	PTT (λex ≈ 800 nm for both formulations) and chemotherapy	[68]
		PVP, PEG, and anti-heat shock protein (HSP) monoclonal antibody	61.2 ± 4.85	−8.2 ± 1.25	N.A.	4T1	4T1 tumor-bearing mice	PTT (λex ≈ 808 nm)	[90]

Abbreviations—PDT: Photodynamic Therapy; PTT: Photothermal Therapy.

**Table 2:** Iron-based metallic-polymer nanoconjugates/nanohybrids, their physicochemical properties, and therapeutic applications (N.D.—non disclosed, N.A.—not applicable).

Metal	Morphology	Modification	Size (nm)	Surface Charge (mV)	Loading	Longitudinal/Transverse Proton Relaxivity	In Vitro	In Vivo	Applications	Ref.
	Ring	GO (graphene oxide) and CREKA (Cys-Arg-Glu-Lys-Ala)	223.3	22 ± 0.4	N.A.	N.D.	4T1 cells	4T1 tumor-bearing mice	MTD and MTT	[6]
Iron	Spheres	HA conjugated with dopamine (HA-DA)	60.7	−16	N.A.	*r*1: 41.3 mM^−1^	A549, HepG2, CT26, B16F10, and 4T1 cells	4T1, B16F10, and CT26 tumor-bearing mice	MRI	[103]
		PLGA, silica, Polyaniline (PANI), and R8-RGD	206	22.8	Cisplatin	*r*2: 258.5 mM^−1^ s^−1^	A549 cells	A549 tumor-bearing mice	PTT (Strong Absorption in NIR region), MRI, and chemotherapy	[104]
		PEG, RGD, D-Glucosamine	32.31 ± 0.71	−30.2 ± 0.76	N.A.	*r*2: 554 mM^−1^ s^−1^	4T1 cells	4T1 tumor-bearing mice	MRI and hyperthermia	[105]
		PEI, PLGA, and HA	159.5 ± 2.3	−9.1	Olaparib (Olb)	Saturation magnetizations: 21.08 emu/g	MDA-MB-231 cells	MDA-MB-231 tumor-bearing mice	RMF and chemotherapy	[106]
		PLGA, gold shell, and Herceptin	285.7 ± 81.4	N.D.	DOX	*r*2: 345.31 ± 23.06 mM^−1^ s^−1^	BT474, MCF, and BT474/Adr cells	BT474 tumor-bearing mice	MRI, PTT (λex ≈ 750–800 nm), and chemotherapy	[107]
		AS1411 and PLGA	201.87 ± 1.60	−10.67 ± 0.25	N.A.	N.D.	MCF-7 cells	MCF-7 tumor-bearing mice	PA/US imaging and PTT (λex = 635 nm),	[108]
		HA-SS-PLA	≈11	N.D.	PTX	N.D.	HeLa cells	HeLa tumor-bearing mice	Chemotherapy	[109]
	Sheets	PDA (polydopamine), and rGO (reduced graphene oxide)	251	−27.5	N.A.	N.D.	MCF-7 cells	N.A.	MRI, PTT (Strong Absorption in NIR region), and PDT	[110]

Abbreviations—MRI: Magnetic Resonance Imaging; MTD: Magnetothermodynamic therapy; MTT: Magnetothermal Therapy; PA: Photoacoustic Imaging; PDT: Photodynamic Therapy; PTT: Photothermal Therapy; RMF: Rotating Magnetic Field Therapy; US: Ultrasound Imaging.

**Table 3:** Copper-based metallic-polymer nanoconjugates/nanohybrids, their physicochemical properties, and therapeutic applications (N.D.—non disclosed, N.A.—not applicable).

Metal	Morphology	Modification	Size (nm)	Surface Charge (mV)	Loading	In Vitro	In Vivo	Applications	Ref.
Copper	Spheres	Lanthanide-doped nanoparticles and PEG	45	N.D.	N.A.	HeLa cells	Cervical cancer tumor xenograft	NIR-II luminescence imaging/CT/MRI, CDT, and PDT	[121]
		p-(OEOMA-co-MEMA)	285	−17.2	TAPP	CT26 cells	CT26 tumor-bearing mice	PA/PI, PTT (Band from visible to NIR), PDT, and SDT	[122]
		DSPE-PEG modified with Lanreotide	186.1 ± 5.2	−16.4 ± 0.1	Docetaxel	PC-3 cells	PC-3 tumor-bearing mice	PA, PI, PTT (Band between 700 and 1000 nm), PDT, and chemotherapy	[123]
		PLGA, PDA, and PEG	288 (Higher MW-PLGA); 257 (Lower MW-PLGA)	−18.7 (Higher MW-PLGA); −22.2 (Lower MW-PLGA)	N.A.	Cal-33 cells	N.A.	MRI, PTT (N.D.), and chemotherapy	[124]
		HA/PEI	330.7	16.9	Disulfiram	Eca109	Eca109 tumor-bearing mice	Chemotherapy and FL	[125]
		PEG-NH_2_ and PCL-SS-P(DPA/GMA/MP)	151.5 ± 2.2	−17.1 ± 1.7	Dox	L929 and 4T1	4T1 tumor-bearing mice	PTT (Strong absorption in the NIR region), and chemotherapy	[126]
		HA and PDA	106	−19.43	Dox	HeLa and 4T1	4T1 tumor-bearing mice	PA, PTT (N.D), CDT, and chemotherapy	[127]
	Framework	Pluronic F127	186.4 ± 16.7	−1.2 ± 0.1	O_2_	4T1 and HeLa cells	4T1 tumor-bearing mice	PDT (Band from visibile to NIR)	[128]
	Cubes	BSA and PEG-FA	60	N.A.	N.A.	HepG2 cells	N.A.	PTT (Band from visible to NIR) and chemotherapy	[129]

Abbreviations—CT: Computed Tomography; PA: Photoacoustic Imaging; PDT; Photodynamic Therapy; PTT: Photothermal Therapy; SDT: Sonodynamic Therapy.

**Table 4:** Summary of the Platinum/Silver/Zinc-based metallic-polymer nanoconjugates/nanohybrids, their physicochemical properties, and therapeutic applications (N.D.—non disclosed, N.A.—not applicable).

Metal	Morphology	Modification	Size (nm)	Surface Charge (mV)	Loading	In Vitro	In Vivo	Applications	Ref.
Platinum	Spheres	PDA and Folate	≈100	N.A.	Indocyanine Green (ICG)	MCF-7	Breast cancer tumor xenograft	PA, FL, PTT (λex ≈ 700–800 nm), and PDT	[136]
		PEG	120	−14.6	DOX	4T1	4T1 tumor-bearing mice	EDT and chemotherapy	[138]
		HA	38 ± 6	−31 ± 1	N.A.	MDA-MB-231 (CD44+) and PC9 (CD44-)	MDA-MB-231 tumor-bearing mice	PI and PTT (N.D.)	[139]
		PEG	119.7	−1.6 ± 0.4	Cisplatin and IR780	4T1	4T1 tumor-bearing mice/Hepatocellular Carcinoma Patient Derived Xenograft	PI, FL, PTT (λex = 780 nm), and chemotherapy	[144]
Silver	Globular irregular shape	BSA and PEG	131.5 ± 2.7	−34.68 ± 0.6	ICG	B16F10 cells	B16F10 tumor-bearing mice	PTT (λex ≈ 790 nm)	[143]
	Spheres	Polythiourea and PEG	25–30	N.D.	N.A.	A549	A549 tumor-bearing mice	FL	[145]
		HA	104 ± 6.2	−30	N.A.	4T1	4T1 tumor-bearing mice	FL and RT	[146]
	Dots	FA modified DSPE-PEG_2000_	200	−30.84	N.A.	HeLa and A549 cells	HeLa tumor-bearing mice	FL/PA imaging and PTT (Strong absorption in the visible and NIR region)	[147]
Zinc	Spheres	PVP40	≈5	−3.6	N.A.	SW480 and HEK293T cells	SW480 tumor-bearing mice	PDT (N.D.)	[133]
		PDA	≈175	−21.7	DOX and DNAzyme	A549 cells	A549 tumor-bearing mice	FL, PI, GT, PTT (N.D.), and chemotherapy	[148]
		PEG and RGD	112.0 ± 3.2	−14.6 ± 5.2	PTX	4T1 cells	4T1 tumor-bearing mice	MRI, NIRFI, and chemotherapy	[149]
		PEG and PLGA	PLGA-ZnNPc-NP = 141; PLGA-ZnPc-NPs = 152	PLGA-ZnNPc-NPs = 4.8; PLGA-ZnPc-NPs = 5.1	N.A.	MCF-7 cells	DMBA-induced breast cancer-bearing mice	FL and PDT (N.D.)	[150]

Abbreviations—CDT: Chemodynamic Therapy; EDT: Electrodynamic Therapy; FL: Fluorescence Imaging; GT: Gene Therapy; MRI: Magnetic Resonance Imaging; NIRFI: Near Infrared Fluorescence Imaging; PA: Photoacoustic Imaging; PDT: Photodynamic Therapy; PI: Photothermal Imaging; PTT: Photothermal Therapy; RT: Radiotherapy.

**Table 5:** Summary of clinical trials comprising metallic-polymer nanoconjugates/nanohybrids (N.A.—not applicable).

Name	Description	Application	Administration Route	Type of Cancer	Clinical Trials Identifier (Phase)	Results	Ref.
Magnablate^®^	Iron oxide nanoparticles	Magnetic Hyperthermia	Intratumoral	Prostate Cancer	NCT02033447 (Early Phase I): Completed	No results yet available	[154]
Nanotherm^®^	Iron oxide nanoparticles	Magnetic Hyperthermia	Intratumoral	Brain tumor	Approved by the EMA in 2010		[37]
			Intratumoral	Prostate Carcinoma	NCT05010759: Still recruiting (Phase not applicable)	No results yet available	N.A.
AuroLase^®^	PEGylated silica-gold nanoshell (AuroShell^®^)	Laser-activated termal ablation	Intravenous	Metastic lung tumors	NCT01679470: Phase not applicable	No results yet available	[156]
				Refractory and/or recurrent head and neck tumors	NCT00848042: Phase not applicable	No results yet available	N.A.
		Laser-activated termal ablation combined with MRI/US fusion technology for focal ablation		Neoplastic Prostate tissue	NCT02680535 and NCT04240639 (extension of the previous): Phase not applicable	No results yet available	N.A.
NU-0129^®^	Spherical gold nanoparticle conjugated with siRNA oligonucleotides	Targeting BCL2L12 oncogene	Intravenous	Glioblastoma multiforme or Gliosarcoma Treatment	NCT03020017: Completed	No results provide about the antitumor efficacy	[158]

Abbreviations—EMA: European Medicines Agency; MRI: Magnetic Resonance Imaging; US: Ultrasound Imaging.

## Data Availability

The data presented in this article are available at request from the corresponding author.
